# Reimbursement for reconstruction by tissue transfer–a European comparison

**DOI:** 10.1186/1472-6963-14-427

**Published:** 2014-09-24

**Authors:** Oliver Lotter, William Arthur Townley, Philipp Gonser, Hans-Eberhard Schaller, Sebastian Hoefert

**Affiliations:** Clinic for Plastic, Hand and Reconstructive Surgery, Burn and Trauma Center, Eberhard-Karls-University, Schnarrenbergstrasse 95, 72076 Tuebingen, Germany; Plastic Surgery Department, John Radcliffe Hospital, Oxford, OX3 8DU UK; Department of Oral and Maxillofacial Surgery, Eberhard-Karls-University, Schnarrenbergstrasse 95, 72076 Tuebingen, Germany

**Keywords:** Tissue transfer, Flaps, Reimbursement, Diagnosis-Related Groups, Health care policy, Health services research

## Abstract

**Background:**

Case payment mechanisms have become the principal means of remunerating hospitals in most developed countries. Our purpose was to analyse the reimbursement for different types of tissue transfer in five European countries.

**Methods:**

We looked at common surgical options for pedicled and free flaps. The recipient site of a flap and the principal diagnosis were systematically modified and processed with national grouper software in order to identify Diagnosis-Related Groups from which the proceeds were derived. The primary data originated from the database of the German Institute for the Hospital Remuneration System as aggregate information. We conducted eight specialist interviews to transfer the available data into clinical practice. Data of real patients were not available and we rather simulated standard patients to avoid dilution of results.

**Results:**

Altogether, payment for pedicled flaps averaged 5933€ and was 8517€ for free flaps. The comparison of both flap types within a country revealed significant differences in Germany, Austria and Sweden only (p < 0.001). Italy has the highest mean proceeds for pedicled flaps, followed by Sweden, Germany, Austria and the UK. This relationship changes for free flaps with Sweden achieving the highest payments. Overall, reimbursement conformity is higher for free flaps.

**Conclusions:**

Most countries have procedure-driven payment systems for flap surgery, which additionally can strongly depend on the diagnosis. Nevertheless the latter does not always justify existing price differences. For the first time, clinical cases in tissue transfer were compared internationally. In today`s dynamic world of health care, we should observe other countries` compensation systems to identify ways of improving our own.

## Background

Reimbursement systems for hospitals are very complex and differ between countries. In this context, Diagnosis- Related Group (DRG) is the most common payment system to classify hospital cases into groups with similar use of resources. Fetter and Thompson created it in 1967 at Yale University [1]. In contrast to fee-for-service payments, capitation fees and per-diem payment reimburses, DRG is a fixed-payment system. The introduction of lump sum reimbursement by DRG has led to profound effects on the hospital landscape in the affected countries. The aims of increasing efficiency, transparency, standardization and cost reduction are barred in particular by fears of quality loss in medical care and revenue declines. Currently, 20 of 34 member countries of the Organization for Economic Cooperation and Development (OECD) have adopted DRG-based payment systems [2]. All countries we looked at adopted this system.

Defect reconstruction through flaps often implies cost intensive and highly elaborate operative therapies using tremendous resources and requiring special technical prerequisites as well as specific microsurgical skills of the operating team. Such therapies are frequently conducted in a multidisciplinary approach. Since the 1970s, free transfer of vascularized tissue has become the procedure of choice in many cases, because this proceed is often able to solve surgical problems more quickly and with less morbidity than traditional techniques [3]. However, increasing concerns about the high cost of such therapies continue to be raised [4]. Furthermore, the difference in pay of the various reconstructive options carries the risk that treatment decisions are influenced monetarily. Overall, these developments lead to an increased utilization of healthcare resources, which must be mastered by national payment systems.

In the following investigation, the German, Austrian, Italian, British and Swedish reimbursement systems are analysed regarding common pedicled and free flaps while considering various underlying diagnosis as well as different recipient regions. Our purpose was to highlight and discuss special characteristics of reimbursement both nationally and internationally. According to our literature review, this study is the first to compare different reimbursement systems according to specific clinical cases in reconstructive plastic surgery.

## Methods

The underlying primary data of our analysis originates from the database of the German Institute for the Hospital Remuneration System of the year 2012 [http://www.g-drg.de/cms/Archiv/Systemjahr_2012_bzw._Datenjahr_2010#sm7]. This database contains aggregate and anonymized data on patients and billing of all German hospitals as well as cost data of selected reference hospitals within the German DRG system (no outpatient services, private inpatient billing or patients within the Employers` Liability Insurance). The aggregated data which is codified by ICD-10- and OPS-Codes (diagnoses and procedures) can only give some information about the localization of flaps but does not allow the identification of a specific flap type. For transfer of the available data into clinical practice, eight specialists in Plastic Surgery with their main activity in Reconstructive Plastic Surgery working at five German, one British and one Italian University Hospital were interviewed. Furthermore, actual versions of known international textbooks were used to identify commonly used flap types with different indications.

Data of real patients were not available nationally or internationally and we refrained from basing our analysis on real patient data of a single institution or a few institutions. However, we expected that some flap operations were performed more frequently than others in our European context, irrespective of personal experience and preferences of decision makers and surgeons in the single hospitals. In this analysis we rather simulated standard patients to avoid irrelevant factors such as comorbidities and complications to dilute the results and drift away from the focus.

We looked at 20 flaps altogether, each 10 pedicled and 10 free flaps with recipient sites on head and neck, trunk (except for the breast), buttocks as well as the upper and lower extremities (Table 1).

**Table 1:** **Allocation of pedicled and free flaps to different recipient areas**

	Flap type	
Recipient area	***Pedicled flaps***	***Free flaps***
*Head and Neck*	Temporofascial flap	Anterolateral thigh flap
	Frontal flap	Free radial forearm flap
*Trunk*	Latissimus dorsi flap	Latissimus dorsi flap
		Gracilis flap
*Buttocks*	Gracilis flap	Latissimus dorsi flap
	Gluteal rotation flap	
	Biceps femoris flap	
*Upper extremity*	Radial forearm flap	Anterolateral thigh flap
	Groin flap	Lateral arm flap
		Serratus fascial flap
*Lower extremity*	Gastrocnemius flap	Latissimus dorsi flap
	Suralis flap	Anterolateral thigh flap

In addition to recipient site modification of a flap, the following matrix for the underlying diagnosis was used to discover possible differences in the DRG-assignment: 1. open wounds, 2. deep lesions with possible co-damage of tendons and muscles, 3. third grade open fractures, 4. infection after operation, 5. osteomyelitis, 6. malignoma, 7. benignoma, 8. full thickness burn injuries. Furthermore, frostbite at hands, feet and nose as well as decubitus ulcers at the head, back, seat area and feet were analysed separately as they are not applicable to all body regions. To simulate different clinical situations, the diagnosis and – if possible – the operative procedure(s) were modified. Each combination of diagnosis and procedure(s) was extended by two additional scenarios (multiple sessions): First, a surgical wound debridement or local excision of pathological tissue was codified in the same setting as the flap surgery. Second, the debridement or local excision was made on day one as a single procedure and repeated five days later together with the flap surgery. This corresponds to a total of 2505 combinations in the 5 different national reimbursement systems (20 flaps × 8 underlying diagnoses × 5 countries × 3 scenarios + frostbite 3 locations × 5 countries × 3 scenarios + decubitus ulcers 4 locations × 5 countries × 3 scenarios).

The diagnoses and procedures were processed to determine the appropriate DRG, using a special grouper software. We applied the German ID Diacos® grouper of Firma ID (Berlin), the official Austrian software package KDok® and the Italian CMS grouper of Fondazione Medtronic Italia® (Milano) Version 24 [5–8]. In the British system, Healthcare Resource Groups (HRGs) were derived from the HRG4 2012/2013 Local Payment Grouper and the reimbursement was then generated by using the national tariff under Payment by Results (PbR) funding policy [9]. For Sweden the identification of NordDRGs was undertaken by the software of DRG System Development AB® (Stockholm) [10]. All groupers used were in the actual version of the year 2012.

Each DRG code maps to a relative weight, which leads to the payment amount for a particular hospital visit. Furthermore, thresholds of length of stay were determined. These trimpoints usually serve as adjustment parameters for medical payments concerning so called outliers where the hospitalisation time exceeds or falls below certain limits. For the consistency of data, we assumed a 50 year old male patient without any complications or relevant comorbidities.

Conversion of Swedish Kronor (SEK) and Great Britain Pound (GBP) into Euro (€) was performed according to the mean exchange rates of the year 2011 (1€ = 9.016 SEK = 0.871 GBP) [11].

Purchasing power parities (PPPs) were used to make payments between the countries comparable. These indicate how many money units are necessary to acquire the same quantity and quality of goods and services abroad as inland [12]. One Euro that has been earned in Germany is worth 6.2% less in Austria, 11.3% less in Italy, 4.4% less in the UK and 4.5% less in Sweden [13].

We used independent sample t-tests to compare the thresholds of length of stay and the reimbursement of the different reconstructive options. The tests indicated significant differences between the reconstructive options if the two-sided p-value was lower than the significance level of p = 0.002. We chose this value to keep the family-wise error rate for the 25 comparisons that we conducted at a 0.05 level (0.05/25 = 0.002).

A Medline literature research included the keywords [(tissue transfer) or (flaps)] and [(Diagnosis-Related Groups) or (reimbursement) or (costs)] and revealed a total of 354 results (status as of 19.06.2012). Out of these, only 18 articles were relevant for our topic.

This manuscript does not report research which requires approval by ethics committee(s) as no human subjects were involved.

## Results

Altogether, 1,920 combinations of diagnosis and procedure(s) for pedicled and free flaps were simulated in the five DRG-systems.

### A. DRGs

Nine different DRGs for pedicled tissue transfer and 7 for free flaps were defined in Germany. For Austria, 3 and 1 DRGs were found respectively and in Sweden 7 and 6 DRGs. In Italy, both types of tissue transfer revealed 6 DRGs each and in England only one single DRG for both pedicled and free flaps was determined.

### B. Length of stay

The upper threshold of length of stay (uLoS) is the only trimpoint available in all countries of our series and is shown in Table 2. The highest values concerning uLoS for pedicled flaps are found in Sweden in combination with burn injuries as the underlying diagnosis (Mean [M] = 47 days, Standard Deviation [SD] = 0 days), followed by deep lesions, infections and burns (M = 35 days each, SD = 0 days) in Italy. Regarding free flaps, again Sweden shows the highest values in burns (M = 47 days, SD = 0 days) followed by open wounds and deep lesions in Germany (M = 41 days, SD = 0 days). Remarkably, some flaps for defect coverage of the upper extremity result in a mean uLoS which is much shorter than for the same flaps transplanted to other body regions. As expected, the uLoS in the five countries is generally shorter for pedicled flaps (M = 20 days, SD = 9 days) when compared to free flaps (M = 25 days, SD = 8 days). Using an independent sample t-test there is a statistically significant difference (p < 0.001) between pedicled and free flaps. When comparing the uLoS between both flap types within the single countries, Italy and the UK do not reach the significance level (p = 0.977 and p = 1.000).

**Table 2:** **Upper threshold of length of stay (uLoS) in pedicled and free flaps (in days)**

	D		AT		I		UK		S	
	***Pedicled***	***Free***	***Pedicled***	***Free***	***Pedicled***	***Free***	***Pedicled***	***Free***	***Pedicled***	***Free***
Open wounds	24	41	14	20	12	12	18	18	10	28
Deep lesions	24	41	14	20	35	34	18	18	13	29
Open fractures	28	29	14	20	23	24	18	18	11	28
Infection	25	38	14	20	35	35	18	18	33	33
Osteomyelitis	21	19	14	20	24	24	18	18	6	27
Malignoma	19	19	14	20	24	24	18	18	6	27
Benignoma	21	19	14	20	24	24	18	18	6	27
Burn injuries	21	30	21	20	35	35	18	18	47	47
**Mean**	**23**	**30**	**15**	**20**	**27**	**27**	**18**	**18**	**16**	**31**
**(SD)**	**(2,75)**	**(9,68)**	**(2,55)**	**(0)**	**(7,96)**	**(7,95)**	**(0)**	**(0)**	**(15,25)**	**(6,89)**

Numbers in brackets indicate one standard error from the mean.

D = Germany, AT = Austria, I = Italy, UK = United Kingdom, S = Sweden.

### C. Reimbursement

**Figure 1:**
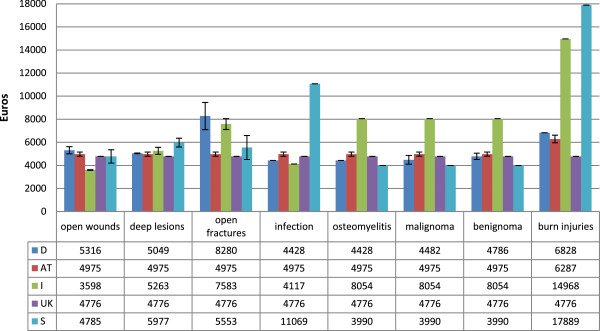
**Mean reimbursement for pedicled flaps with subdivision into the underlying diagnosis.** Error bars indicate one standard error from the mean. D = Germany, AT = Austria, I = Italy, UK = United Kingdom, S = Sweden.

**Figure 2:**
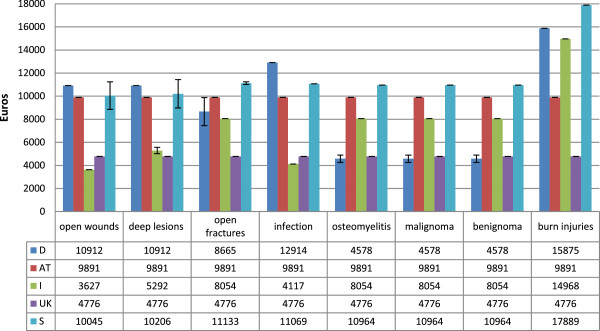
**Mean reimbursement for free flaps with subdivision into the underlying diagnosis.** Error bars indicate one standard error from the mean. D = Germany, AT = Austria, I = Italy, UK = United Kingdom, S = Sweden.

The single values of reimbursement for pedicled and free flaps are shown in Figures 1 and 2. Italy has the highest mean proceeds for pedicled flaps (M = 7461€, SD = 3376€), followed by Sweden (M = 7155€, SD = 7155€), Germany (M = 5450€, SD = 1898€), Austria (M = 5139€, SD = 761€) and the UK (M = 4776€, SD = 0€). This relationship changes for free flaps with Sweden achieving by far the highest reimbursement (M = 11654€, SD = 3003€). Proceeds for pedicled flaps with osteomyelitis in Sweden (M = 11069€, SD = 0€), as well as burn injuries of all body regions in Italy (M = 14968€, SD = 0€) and Sweden (M = 17889€, SD = 0€) are in a five-digit range. For free flaps, this is the case most commonly found in Germany (range 10912€ - 15875€) except for open fractures, osteomyelitis and tumors. Again, Italy shows one of the highest reimburses for free flaps in burns (M = 14968€, SD = 0€) and Sweden has all of its flaps reimbursed by five-digit numbers (range 10045€ - 17889€). Generally, there is only a slight difference between both types of tissue transfer in Italy. It is noticeable that both flap types in open fractures, osteomyelitis as well as benign and malignant tumors lead to much lower reimbursement in Germany when the recipient site is the upper extremity (all M = 3119€, SD = 0€) compared to the other body regions. Proceeds in Austria are very constant except for pedicled flaps in burn injuries for which more is paid. In Sweden the type of operation mainly determines the payment and the underlying diagnosis only plays a minor role. In general, proceeds are highest for defect reconstruction after full-thickness burns in all analysed countries.

The mean value of the five countries is 5933€ (M range 2159€ - 17554€, SD = 2987€) for pedicled flaps compared to 8517€ (M range 3119€ - 17554€, SD = 3600€) for free tissue transfer. Significantly higher values were paid for free flaps than for pedicled flaps (p < 0.001). The comparison of both flap types within a country revealed significant differences in Germany, Austria and Sweden only (p < 0.001).

### D. Multiple sessions

When looking at the scenarios of multiple sessions, only Austrian proceeds increased. However this was related to benign and malignant tumors as well as full-thickness burns. Regarding scenario I (surgical wound debridement or local excision in the same setting as the flap surgery) the average increase amounted to 19% in pedicled and 10% in free flaps. For scenario II (debridement or local excision on day one and repeated five days later together with the flap surgery), additional proceeds of 36% or 21% could be found. The additional temporary wound closure such as Negative Pressure Wound Therapy did not reveal any changes in the definition of DRGs.

### E. Special situations

The reimbursements for frostbite and decubitus ulcers are shown in Table 3. In Italy and the UK, there is no difference between the two categories of flaps. The Austrian system shows no variation regarding other underlying diagnoses. A separate DRG for decubitus ulcers is available in Germany, however it does not distinguish between pedicled and free flaps. The highest discrimination can be found in Sweden where different DRGs exist for pedicled and free flaps as well as for decubitus ulcers and frostbites.

**Table 3:** **Reimbursement for special situations (in Euros)**

a) Frostbite					
	D	AT	I	UK	S
Pedicled flaps	5010	5160	5708	4578	6798
Free flaps	10912	9891	5708	4578	12417
**Mean**	**7961**	**7526**	**5708**	**4578**	**9608**
**(SD)**	**(4173)**	**(3345)**	**(0)**	**(0)**	**(3973)**
**b) Decubitus ulcers**					
	**D**	**AT**	**I**	**UK**	**S**
Pedicled flaps	8702	5160	4117	4578	10601
Free flaps	8702	9891	4117	4578	12614
**Mean**	**8702**	**7526**	**4117**	**4578**	**11608**
**(SD)**	**(0)**	**(3345)**	**(0)**	**(0)**	**(1423)**

Numbers in brackets indicate one standard error from the mean.

D = Germany, AT = Austria, I = Italy, UK = United Kingdom, S = Sweden.

### F. Single-case comparison and purchasing power parities

**Figure 3:**
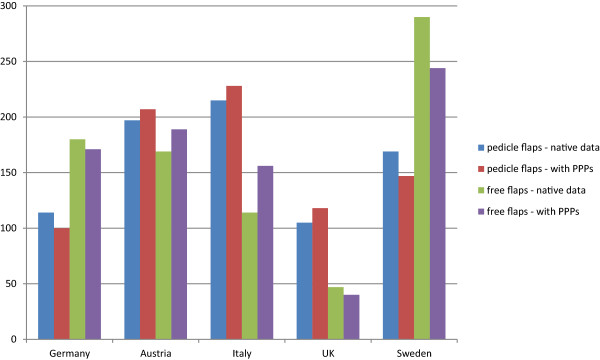
**Single-case comparison of reimbursement between the countries** **.** The reimburses of the different categories of all countries are compared among each other. There are a total of 8 underlying diagnoses in each 10 pedicled and 10 free flaps in every country. Thus, 160 scenarios are compared between two countries (8×10×2) with 10 different country pair formations.

All single-case reimbursments out of the different countries were compared with each other regarding the specific flap used. Altogether 20 different flap types, each 10 pedicled and 10 free flaps, as well as 8 underlying diagnoses were compared from the five above mentioned countries. This led to 1600 direct, single-case comparisons of reimbursement for flaps (Figure 3). Furthermore, Purchasing power parities (PPPs) were used to improve the international comparability of the proceeds. When comparing the native data of pedicled flaps, Italy has most of the highest reimburses, followed by Austria, Sweden, Germany and the UK. This relationship changes when integrating PPPs, placing the UK at the fourth position, thus overtaking Germany. Concerning reimburses of free flaps, Sweden is in the first place, followed by Germany, Austria, then Italy and finally the UK. Austria overtakes Germany when PPPs are applied.

## Discussion

In the present investigation the German, Austrian, Italian, English and Swedish reimbursement systems were compared regarding surgical treatment through pedicled and free flaps with different underlying diagnoses in various body regions. Our aim was to standardize the scenarios to ensure maximal transparency and comparability. Therefore, a predefined algorithm to depict the various tissue transfers both nationally and internationally was applied. We were surprised to find salient contrasts in thresholds of length of stay and reimbursement.

Altogether, we found statistically significant lower values for the upper length of stay (uLoS) for pedicled flaps than for free flaps. In this context, the authors would like to underline the importance of the lower threshold (lLoS), which can only be found in Germany and Austria. Systems with no lLoS have to be critically judged as encouragers of “bloody discharges” and bear the risk of quality loss in medical treatment.

We assumed the reimbursement for free flaps to exceed that of pedicled flaps. This was the case for the average remuneration in Germany (9126€ vs. 5450€), Austria (9891€ vs. 5139€) and Sweden (7036€ vs. 11464€) with statistical significance. Interestingly this discrepancy was found to be much lower in Italy (7461€ vs. 7527€). Furthermore in the UK reimburses for both flap types showed no difference (both 4578€). For pedicled flaps, the mean value for reimburses of the five countries was 5933€ and in contrary 8517€ for free tissue transfer. We found no different procedure codes to distinguish free myocutaneous flaps from perforator flaps such as for reconstructive breast surgery in some countries [14]. The authors think this would also make sense for tissue transfer to other body regions than the breast, however, no information about potential cost differences is available so far that could justify such a distinction.

Overall, in reconstructive surgery the British and Austrian systems strongly depend on the procedure(s) and the underlying diagnosis is virtually of no importance. In contrast, grouping in Italy relies on the diagnosis but – as in the UK - there is no adequate discrimination between pedicled and free flaps. The German system is mainly driven by the underlying diagnosis and less by the procedure(s). It has to be questioned whether the leading diagnosis is supposed to have such a strong effect on reimbursement especially in free flap surgery. One exception might be the treatment of burns where the multimodality and complexity of treatment is often higher than in other cases.

Our extensive literature research revealed that most publications concerning flaps were available in the field of Head and Neck, Oromaxillofacial and Breast Surgery and only a few were related to other body regions [15–31]. All articles we found were published in medical journals and none in other fields such as health economics or health services research. Interestingly, all but two papers were exclusively analysing the costs of tissue transfer, usually comparing methods of pedicled with free flaps. In this respect, cost-identification analyses were by far the most common type [32]. Differences between pedicled and free tissue transfer were shown to be closely related to length of operative procedure and its subsequent impact on postoperative care including longer stays in the intensive care unit. Mainly in the field of Oromaxillofacial Surgery, other outcome variables showed no statistically significant difference [15–19]. In these studies, the reconstruction type was not significantly associated with overall costs in the majority of cases. We were surprised about these findings, as free tissue transfer requires additional technical equipment (operation microscope), special surgical skills (microsurgical training), mostly longer operation times and higher requirements concerning aftercare and monitoring.

The two papers considering reimbursement were authored in Anglo-American countries with just one analysing two different flap types (pedicled vs. free flaps) and none examining different body regions [22, 31]. Furthermore our literature research revealed that all studies based their analysis only on data of one national institution, which by far has no claim to universal validity. For the first time we compared payments internationally avoiding a single-center approach and trying to maximize standardisation between the countries.

One-stage procedures with debridement or tumor resection and tissue transfer during the same hospital stay were considered separately. The higher resource use in these cases compared to reconstruction by flaps only seems logical but reimburses usually remain the same without considering earlier operative procedures during the same stay and reimbursing these cases higher. Unfortunately, up to now there are no cost-identification analyses available to point out this problem.

This study is limited through the focus on only the main diagnosis to create standardised “case vignettes” avoiding comorbidities and complications to ensure comparability and reduce complexity. The selection and distribution of the various flaps was randomly and will strongly depend on the spectrum of a single department performing reconstructive surgery. Furthermore, comparison of international DRG-related data was impaired by numerous variables such as different structural conditions, organization and other factors that cannot be taken into account. The healthcare market is still highly regulated and free market forces are often underrepresented which could lead to falsification [33]. As in most cost analyses, where the primary focus has been identification of costs incurred during the initial hospitalization in which a flap procedure was performed, we also concentrated on reimbursement for the first hospital stay. Consecutive operations were excluded. Last but not least, Purchasing Power Parities allowing international comparison depict the situation of a predefined consumer basket and might not exactly describe the focus of the health care sector.

It would be interesting to include the cost of each scenario to compare the profit of different tissue transfers within and among the countries. However, cost data – if available – often shows less consistency and a large range throughout a country than reimbursement. The mixture of completely different treatment cases under the cover of DRG, resulting in a different consumption of resources is a major barrier for a transparent cost presentation. Therefore we recommend in a first step to exclude the diagnosis from the calculation of reimbursement except for highly elaborate pathologies such as burns for example. In doing so, more emphasis could be put on the operation, which seems to be the main trigger of costs in tissue transfer. In this context, however, monetary reasons are not allowed to motivate the surgeon for or against certain specific therapies.

## Conclusions

Flap surgery is necessarily individualized and specific procedures may be indicated in very different conditions and circumstances, each with a unique pattern of resource use and often independent of the underlying diagnosis. As pointed out in our analysis, this is in contrast to many rigid and inflexible coding systems grouping different flap procedures under only one single procedure code [30]. This leads us to the question whether there should be a higher reimbursement discrimination between various reconstructive options in cases where future in-depth cost analyses have shown obvious discrepancies. For the first time, clinical cases in tissue transfer were compared internationally. The comparison of reimbursement by DRG should be a useful instrument for benchmarking and refinement of a national compensation system while not depending solely on political decisions or country-specific cost data.

## Abbreviations

CMS: Centers for Medicare and Medicaid Services
DRG: Diagnosis-Related Group
FDA: Food and drug administration
GBP: Great Britain Pound
HCFA: Health care financing administration
HRG: Healthcare resource group
ICD-10: International classification of diseases
LD: Latissimus Dorsi
NHS: National Health Service
OECD: Organization for Economic Cooperation and Development
PbR: Payment by results
PPP: Purchasing power parity
SEK: Swedish Kronor
TRAM: Transverse rectus abdominis muscle
uLoS: Upper Length of Stay.
